# Contract Design: The problem of information asymmetry

**DOI:** 10.5334/ijic.3614

**Published:** 2018-01-12

**Authors:** Axel C. Mühlbacher, Volker E. Amelung, Christin Juhnke

**Affiliations:** 1 Health Economics and Healthcare Management, Hochschule Neubrandenburg, Neubrandenburg, DE; 2 Institute of Epidemiology, Social Medicine and Health System Research, Hannover Medical School, Hannover, DE

**Keywords:** contract design, integrated care, information asymmetry

## Abstract

**Introduction:**

Integrated care systems are advocated as an effective method of improving the performance of healthcare systems. These systems outline a payment and care delivery model that intends to tie provider reimbursements to predefined quality metrics. Little is known about the contractual design and the main challenges of delegating “accountability” to these new kinds of organisations and/or contracts. The research question in this article focuses on how healthcare contracts can look like and which possible problems arise in designing such contracts. In this a special interest is placed on information asymmetries.

**Methods:**

A comprehensive literature review on methods of designing contracts in Integrated Care was conducted. This article is the first in a row of three that all contribute to a specific issue in designing healthcare contracts. Starting with the organisation of contracts and information asymmetries, part 2 focusses on financial options and risks and part 3 finally concludes with the question of risk management and evaluation.

**Results:**

Healthcare contracting between providers and payers will have a major impact on the overall design of future healthcare systems. If Integrated care systems or any other similar concept of care delivery are to be contracted directly by payers to manage the continuum of care the costs of market utilisation play an essential role. Transaction costs also arise in the course of the negotiation and implementation of contracts. These costs are the reason why it is generally not possible to conclude perfect (complete) contracts. Problems with asymmetric distribution of information can relate to the situation before a contract is concluded (adverse selection) and after conclusion of a contract (moral hazard).

**Discussion and Conclusions:**

Information asymmetries are seen as a major obstacle to the efficient operation of integrated care programmes. Coordination and motivation problems cannot be solved at no-costs. The presented problems in the design of selective individual contracts represent a necessary but not a sufficient condition for further government intervention. A state or political failures have to be assumed continuously.

## Background and Objective: How to design healthcare contracts and how to deal with asymmetric information?

Accountable care organisations (ACOs) in the US and similar concepts in other countries are advocated as an effective method of improving the performance of healthcare systems [1]. ACOs outline a payment and care delivery model that intends to tie provider reimbursements to predefined quality metrics. By this the total costs of care shall be reduced [2].

Little is known about the contractual design and the main challenges of delegating “accountability” to these new kinds of organisations and/or contracts. The costs of market utilisation are highly relevant for the conception of healthcare contracts; furthermore information asymmetries are an obstacle to the efficient operation of ACOs [1].

A healthcare contract is a relational contract, which determines the level of reimbursement, the scope of services and the quality between service providers and payers, taking account of the risks relating to population and performance. A relational contract is an agreement based upon assumption of a longer timeframe [3,4]. Upon conclusion of the contract only a framework is agreed, the specific details are only finalized over the course of the agreed contractual period.

Healthcare contracting between providers and payers will have a major impact on the overall design of future healthcare systems [1].

### The Healthcare Market: Segmentation of Property Rights

If Integrated care systems, ACOs or any other similar concept of care delivery are to be contracted directly by payers to manage the continuum of care the costs of market utilisation play an essential role. Contrary to neoclassical theory, the transaction cost approach [5] assumes that the market does not operate without costs. Transaction costs also arise in the course of the negotiation and implementation of contracts. These costs are the reason why it is generally not possible to conclude perfect (complete) contracts. The property rights theory however focuses on the individual behaviour of the actors, which is influenced by the way in which the property rights are distributed. One possible solution for the basic problem of motivation is the distribution of the property rights to an object or person among various interacting individuals with their own interests [1]. Every healthcare contract can be seen as a division of property rights.

### Problems with the segmentation of property rights

**Figure 1:**
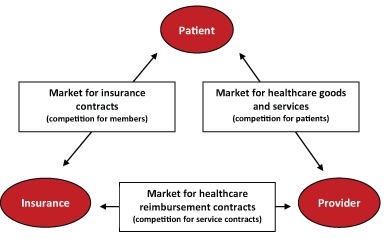
Healthcare Markets (own figure).

The contracting parties on each of the three healthcare markets (Figure 1) are free to allocate each party a certain bundle of property rights, i.e. shares in entrepreneurial success or failure. A healthcare reimbursement contract transfers property rights over the human capital or nonhuman assets of medical care services and facilities the health insurer in order to provide healthcare services defined in more detail in the contract. After conclusion of the contract, the integrated care organisations supply their knowledge, their time, drugs, medical technology and the necessary aids and remedies in the service of the health insurer. In return they receive remuneration. The medical insurer has the right to request performance of the healthcare services specified in the healthcare contract or the care of a body of members defined in advance for the agreed healthcare period. The basic conflict arises from the different goals. Due to the increasing competition, service providers are striving to achieve maximal financial exploitation of their property rights. The health insurers, as custodians of the monies provided by their members, want the best possible services for the lowest possible price.

## Methods

**Figure 2:**
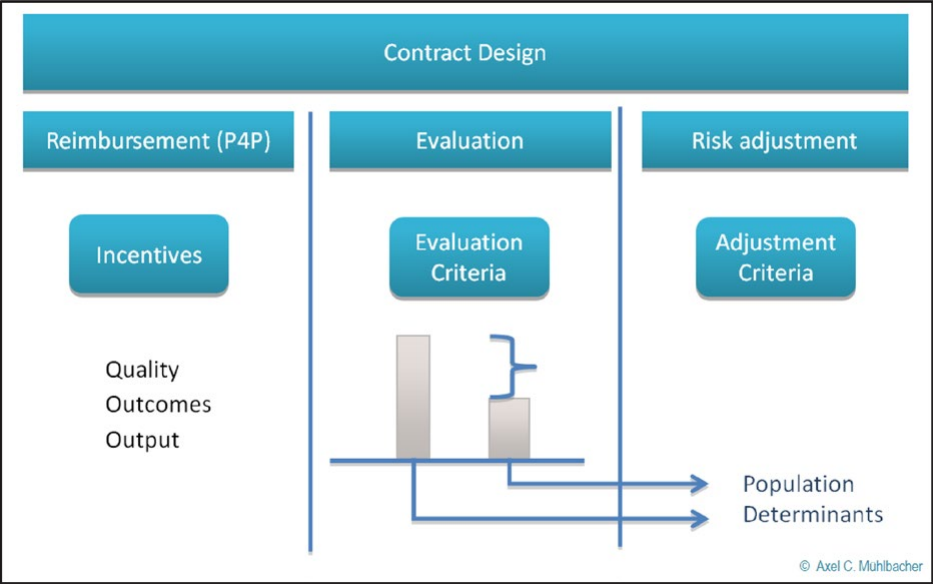
Reflections on the meaning of contract design (own figure).

The research questions in this series of three articles focus on how reimbursement strategies (especially Pay for Performance P4P), evaluation of measures and methods of risk adjustment can best be integrated in healthcare contracting while taking care of specific problems at the same time. The following figure (Figure 2) displays the first reflections on the underlying structure in the topic of healthcare contracting. As depicted contract design influences and is influenced by three main components, financing and reimbursement, risk adjustment and finally evaluation and controlling of risks as well as other contractual issues. This series article will spot light on these components.

In order to answer the research questions for this series of articles a descriptive study was performed based on a comprehensive literature review on methods of designing contracts in Integrated Care. This was conducted as part of the project “International Research on Financing Quality in Healthcare – Interquality” between 2011 and 2014.

## Contract Design: Possible options and possible barriers

### Basic models of contracts

#### Co-operation model

In a co-operation model the health insurance concludes integrated care contracts with various care providers that include a description of services and rule the attributable compensation components for each provider. The care providers are organized among themselves in the form of cooperative agreements without corporate bonds. Each care provider remunerates those integrated care services allotted to him with the insurer. The main controlling and administration tasks are handled by the insurance provider [6,7].

#### Accountable care organisations

(ACOs) are seen to be an effective method of improving the performance of the US healthcare system. The fragmentation of healthcare provision should ideally be abolished in favour of coordinated care and an integrated care model. The goal of ACOs is to pay providers in a way that encourages them to provide coordinated services, does not encourage supplier induced demand, and to create an integrated organisation that is rewarded for providing high quality care. This aim is on the healthcare reform agenda since many years [8,9] but little have been achieved. We believe that a lack of understanding of the main challenges’ of selective contracting and the required institutional regulations is decisive for the little improvements during the last decades.

ACOs could take various forms [10], but they have generally been thought of as groups of primary care physicians, specialists, and sometimes hospitals, joined together in either vertically integrated systems or networks. These networks are eligible for financial bonuses if performance goals are met. Therefore this concept aims at single long-term contracting and building of partnerships between groups of healthcare providers that join forces, with or without hospitals, to form legal entities that agree to take responsibility for the quality, cost and is intended to improve overall care of a population of patients. But the division of work within integrated care systems requires coordination. In theory the market is an efficient coordinating instrument, but only in the absence of transaction and information costs. The costs of market utilisation are relevant for the conception of healthcare contracts and the development of accountable care organisations [1].

### Assessment and Organisation of contracts

#### Selective contracting

The principal items are selective contracts between payers and groups of healthcare providers that join forces, and form legal entities that agree to take responsibility for the quality and cost of healthcare services for a defined (sub-)population for a defined period of time. The high-put target is to improve overall care of a population of patients [1,11].

A selective contract or individual contract is a contract of one party with a single counterparty. Contrary to a collective agreement it offers the opportunity to single providers to individually negotiate terms of the contract with the health insurance. Selective contracting means that a payer is not forced to accept the charges for the use of any physician, hospital or nursing facility by the insured. Rather, only the services of providers are reimbursed, who have entered into a contract with the insurance provider. Through selective contracting, healthcare cost should be controlled, quality assured and planning certainty assured [11].

Essential feature of the legal provisions relating to integrated care in most countries is the option of concluding individual contracts. This means that an insurance company may, either alone or in conjunction with other health insurance companies contract with one or more providers. In terms of integrated care the principle of contractual freedom plays an important role. Providers are not entitled to conclude an integrated contract. It is more likely to assert themselves through innovative ideas and concepts relating to the conclusion of an integration contract over its competitors in the healthcare market.

However, due to the open wording of the legislature contracting parties are allowed to set new priorities in healthcare delivery, on the other hand they have to negotiate every detail individually [11].

##### Criticism on selective contracting

The disadvantage of such individual contracts lies in the heterogeneity and complexity of the healthcare delivery. There is a risk that a nationwide and comprehensive transition to individual contracts could result in greater deviations of quality of medical services and higher transaction costs than in the collective system.

In addition, through direct contracts, a targeted selection of the care provider and a particular care provision (indication) can be made. This means that contracts are only concluded with the groups of providers that offer more performance, more quality, more amenities and/or lower prices [12].

This results in two levels of competition. On the first level the health insurance requests care provider for their network according to certain selection criteria. On the second and lower level the patient decides for certain providers. The focus on the first level, which does not apply to collective bargaining agreements, is the mutual competition of health insurance companies and healthcare providers for contracts and contractors. At the downstream level care providers compete for patients. The selective contracting causes a promotion of efficient and innovative forms of care through more market-based incentives. The “common and unified negotiating” in healthcare is abandoned and “free bargaining” is enabled. Thus, health insurance companies and provider groups can differentiate themselves from their competition through the actual product offerings [11,12].

However, in this selection or non-selection of specific providers risk selection is also made possible. There seems to be certain chances for health insurance companies not take those providers under contract whose clients represent bad risks [13].

For the first time a specific exclusion of certain risks is possible through the design of care contracts. Another disadvantage in selective contracting is the restricted freedom of choice of the insured. Within selective contracts the freedom of choice is limited to care providers selected by the payer with respect to a lower-cost care. Payers hope to obtain better cost control while maintaining or possibly even improve quality of care [13].

#### Contractual items: Asymmetric information

Specific investments play a role in healthcare reimbursement contracts because contractual conclusion and contractual performance do not take place at the same time. The contractual partners have incomplete information since utilisation over the contractual period in accordance with the morbidity and risk factors cannot be established ex-ante. The theory of incomplete contracts is based on the assumption that the contracting partners are in fact able to observe the behaviour and actions of other contracting partners, but that it is not possible for either of the parties to verify the behaviour to an external third party. This also includes verifiable and contractible contractual components which are characterized by the fact that contractual conclusion can only be realised with prohibitively high costs.

If one of two parties has an information advantage, this can be used by opportunistic behaviour to the disadvantage of the other party as part of the contractual relationship. This option for exploitation exists if the parties are unable to agree on an incentive-compliant conditioning of service and reward in the contract [7].

### Problems with asymmetric distribution of information: Adverse Selection and Moral Hazard

#### Problems before contractual conclusion

Problems with asymmetric distribution of information can relate to the situation before a contract is concluded (adverse selection [14]) and after conclusion of a contract (moral hazard). *Hidden characteristics* are attributes of the contracting partner or the subject-matter of the contract which are concealed from the principal ex-ante but revealed ex-post. The networks will attempt to convince the health insurer before contractual conclusion of the quality of both the service and the organisation. In this process faults and weaknesses are concealed in order not to endanger conclusion of an advantageous contract. This results in the risk of adverse selection and means that for the agent there is incentive to display opportunistic behaviour ex-ante, for the principal there is a risk of selecting an unsuitable contractual partner/contractual subject-matter. It is not possible for the principal to discover the intentions of the agent ex-ante, i.e. the aspects of goodwill, fairness and honesty are not known or not adequately assessable before contractual conclusion (*hidden intention*). The intentions of the contractual partner before contractual conclusion form the basis for the problem of *hold-up* which is analysed in the theory of incomplete contracts (see above). The problem of *hold-up* affects both payers and service providers: both sides have an interest in the extent to which the contractual partner will try to opportunistically exploit a chance to take advantage [6].

#### Problems after contractual conclusion

It is often not possible ex-post to draw conclusions about the level of effort invested by the agent on the basis of the result (*hidden action*). Moreover, the result also depends on other exogenous factors. This means that the principal knows the result of the action, but does not know what part the agent played in this result and how much was due to exogenous (environmental) factors. An extreme form of this information asymmetry is described by the term *hidden information*. This means that, although the principal is able to observe the actions of the agent after contractual conclusion, he cannot adequately evaluate them (e.g. on account of the lack of specialist knowledge). This asymmetry of information can be exploited by the agent. In an analysis of contractual negotiations the problem of *hidden information* plays a significant role. The issue arises especially if the information asymmetry is particularly great due to the medical expertise of the service providers. The service providers may after contractual conclusion either have an information advantage (*hidden information*) or be able to exploit the opportunity to act in concealment (*hidden action*). As a result, incentive for opportunistic action arises. The service providers will then pursue their own goals and not act in the interests of the health insurer. The presentation of a case for treatment forms the boundary between two possible expressions of moral hazard (analogous to patient behaviour [15: 208]). After contractual conclusion up to presentation of a case for treatment, the issue of moral hazard may arise “ex-ante”, i.e. the contracting partners fail to adequately influence the probability of a need for treatment through comprehensive precautionary and preventative measures. After presentation of a case for treatment, the medical care services are uncertain in that there are a variety of treatment options available which may be administered. There must be no withholding of services on the basis of individual financial implications. On a market with complete market transparency the problems of adverse selection and moral hazard described above do not occur. This means that measures which increase transparency or contribute to the reduction of information asymmetry also contribute to solving these problems [6].

### Contractual items: reduction of information asymmetries

#### Activities before contractual conclusion – signalling, screening and self-selection

The principal can increase market transparency through *screening*, i.e. direct searches for information [16]. He can, for instance, reduce the information asymmetries in comparison to potential contracting partners by tests, by obtaining several offers and by checking up on reputation and references. The term *screening* describes all information actions aimed at preventing the principal from selecting an unsuitable agent or an unsuitable offer. Information asymmetries can however also be reduced by the agent itself. The term *signalling* describes all activities of the agent which contribute to credibly signalize to the principal the characteristics of the agent [17]. In general, recommendations, references, certificates or guarantees can increase the transparency of the agent’s characteristics. By way of an appropriate range of contracts, payers can achieve *self-selection* of service providers. In terms of contract risks, guarantee claims and forms of remuneration it can be assumed that good suppliers and bad suppliers will select different contracts [6].

##### Screening, signalling and self-selection measures involve costs

The value of a measure depends on the cost of screening or how expensive it is to imitate positive signalling. Individual health insurers who conduct a screening of service providers must assume that the cost of these measures exceeds the benefit derived from reducing the information asymmetry. The publication of information on the quality and particular competence of service providers in the healthcare system serves transparency. Service providers will not stop sending “false signals” until the costs are higher than the benefit of the “false” signal. In the case of guarantees, the costs of providing poor service must be higher than actually ensuring the provision of high-quality healthcare. In addition, suppliers of healthcare programs can undergo voluntary certification. A large majority of health plans providers in the US obtain certification from the evaluating body HEDIS (Healthcare Effectiveness Data and Information Set) of the National Committee for Quality Assurance (NCQA). These instruments and procedures for the external quality assurance of *Managed Care Organisations* (*MCOs*) are offered by the National Committee for Quality Assurance. The goal of external quality assurance is to improve the quality of care by evaluating service providers and their quality of care and by appropriate reporting of the results. The aim is certification and presentation of healthcare quality on the basis of a standardized catalogue of structural, procedural and outcome indicators. If in the future selective contracting or increased establishment of integrated care networks should lead to a strong differentiation of health insurers then – complementary to the quality management within these organisations – measures of external quality assurance and an increased transparency of care in the system of healthcare provision should be implemented [6].

#### Activities after contractual conclusion – monitoring and reporting

After an agreement is contractually established there is a risk of opportunistic behaviour within the scope of *hidden action* and *hidden information*. Observation of the agent by the principal is called *monitoring*. These activities can consist of introducing a common quality management system or introducing medical care planning and control systems on the basis of jointly agreed clinical pathways as well as disclosure of the cost settlement and accounting systems. Utilisation management is used to monitor and steer provision of services on the basis of treatment guidelines, resulting in an overview of the whole healthcare provision process. The goal is comparison of medical care services with target criteria or the comparison of several service providers in the form of benchmarking. *Monitoring* does however require investment of financial resources and time. Delegation of these supervisory measures creates another principal/agent conflict since it results in third parties acting as agents and therefore also requiring supervision. In this context it seems advisable to involve competitors of the contractually established networks in the monitoring since, under competition conditions, they have an interest in uncovering the faults and weaknesses of the agent. If the service provider organisations have an interest in long-term contractual relations, they will also collaborate in reducing information transparency [1,6].

Even after contractual conclusion they will disclose their decisions and actions to the payers. Documentation of activities by the agent itself is known as *reporting*. Service providers can compile reports on the medical care services provided and inform the health insurers of the decisions and therapy steps undertaken. It is also possible to involve the health insurers in decisions and undergo voluntary checks. The concept of external quality assurance is acquiring increasing significance. However, it should also be pointed out that both the acquisition of quality management certification and the maintenance of a quality management system can generate high internal and external costs. Service providers may have an increased interest in reporting measures if they have to assume that the payer is often not able to distinguish whether a medical care service has been performed badly or whether its poor quality was the result of external factors over which the provider has no control. It is then in the interests of the service provider to present evidence of the efforts made to achieve a positive result [7].

### Problems in designing health care contracts

#### Unforeseeable circumstances and framework conditions

Problems arise from changes in the contractual situation due to unforeseeable framework conditions. Although potential changes are foreseeable on account of the risks of a healthcare contract, they cannot be specified in detail in a contract. The concept of the relational contract provides for the fact that the contracting parties – due to prohibitive transaction costs and future contingencies – deliberately leave “gaps” in contractual concepts and agree instead explicitly or by implication procedural rules which allow renegotiation. There is in effect no contract on integrated care which contains regulations for all future contingencies and environmental circumstances. Most importantly the interest in adapting the contracts is mutual and socially unavoidable. Due to the specific characteristics of healthcare goods, all would not accept sticking to contracts: the government, the patient and the contract partners. In the case of an unforeseeable eventuality it cannot be assumed that the contracting parties will display incentive-compatible behaviour. The result of healthcare programs depends on many factors. The consequence of this is that evaluation of the quality of services provided is often characterized by an inability to accept liability or impose sanctions [7].

#### Hold-up and renegotiation

Complete contracts, that are agreements which in advance establish the services to be performed by the contractual partners in all environmental circumstances, cannot be concluded. The conclusion of healthcare reimbursement contracts – particularly with specific investment for the establishment and maintenance of innovative forms of care – cannot be simply assumed in such a context. Health insurers and service providers are aware that due to changing framework conditions, renegotiations are possible. Depending on the level of the specific investment, one of the two contracting parties can be more strongly committed to the contractual relationship (*lock-in*). This means that the investor becomes dependent upon his contractual partner (e.g. a new lab established just for a contract and useable for something else). Ex-post opportunistic behaviour (*hold-up*) leads to the contracting party with the lower specific investment threatening to breach the contract, particularly if the contract is not renegotiated in his favour. The mere expectation of renegotiation has an effect on the making of a specific investment. In the healthcare industry a significant number of actors are non-governmental organisations with complex corporate targets and strongly linkage the local community. Therefore cancelling a contract is often simply not an option [7].

#### Underinvestment and barriers to innovation

Service providers (contractors) and health insurers (clients) make contract-specific investment only on condition that they can expect higher yields in the specific contractual situation. Following failure of the contractual relationship, the value of an investment cannot be realised by sale, i.e. the costs cannot be recouped (*sunk costs*). The opportunistic behaviour “robs” the contracting partner with the larger specific investment of the quasi-rent. A quasi-rent can only be achieved by the service providers if specific investment in cooperation with a payer or in a healthcare offer represents a greater value than outside a healthcare reimbursement contract. The investing partner will anticipate the risk of a *hold-up* and cause loss of prosperity by underinvestment. This underinvestment can be interpreted as a barrier to innovation in the establishment of innovative forms of healthcare: investment is not made in research and development or in the use of innovative technologies (such as information technologies) and forms of therapy concepts (nether the less as physicians belongs to independent occupations the leeway is limited to exclude new methods and especially new drugs). An analysis should therefore be undertaken of the conditions under which efficient governance structures can be specifically invested “optimally” [1].

### Solutions for the problems in the designing of healthcare contracts

#### Vertical integration of contracting partners

From the theoretical and non-healthcare related viewpoint of the (new) theory of property rights postulated by Grossman/Hart [18], Hart/Moore [19,20] and Hart [21], one possible solution for the hold-up problem or for threatened underinvestment is the vertical integration or implementation of complete contracts. Assuming positive transaction costs, no “first best solution” in a neoclassical sense is to be expected without integration or implementation of a three-way control (including a mediator) [5]. A contract which takes account of all eventualities assumes perfect information and flawless rationality. However, this is an idealistic and therefore unrealistic concept, not only in the healthcare system. Formulation of a complete contract would also be a costly enterprise, especially as all the future uncertainties cannot be estimated in total. Particularly in the healthcare system, investment in innovative forms of care involves high levels of capital or is linked to specific indication-linked treatment programs or particular institutions (large health insurers). Moreover, many investments in healthcare technologies, co-operations or innovative therapy and healthcare programs are irreversible to a large extent. The hold-up phenomenon contributes to the fact that often the amortization level of the investment is not reached and thus there is no ex-ante incentive for the actors to invest. As described above, the solution for this problem lies in standardisation of the control rights. This depends on the assumption that ownership is a clearly defined decision right and can be implemented free of charge. The contracting partner who is at risk of hold-up receives the residual decision rights. In the case of integration he becomes the owner of the contracting partner’s enterprise; his residual decision right is limited to the decision as to whether and to what extent investment should be made in human or non-monetary assets. Only then are the profits of the healthcare reimbursement contract can be realised. In the case of vertical integration there is a superordinate management instance. This creates the possibility of complete internalisation of the yields from specific investments. The contracting party with the lowest specific investment, i.e., with the exploitation option, should sell the production factors to the enterprise with the risk of hold-up. A comprehensively integrated managed care organisation (MCO) is on the one hand able to generate synergy effects, but on the other hand it also holds risks, e.g. through the exploitation of discrimination potential. It can be assumed that where there is discrimination potential, there will also be discrimination: on account of specific financial considerations, patients and insurance fund members receive, e.g., no or insufficient services [7].

#### Long-term contracts

Even if the integration of health insurers is not under discussion at the moment, it makes sense in terms of removing the problem of opportunism or the problem of underinvestment to conclude long-term contracts between the relevant health insurers and service providers. Under the condition of significant uncertainty, long-term contracts lead to the fact that specific investment in the development and reputation of a contractual partner present no incentive for hold-up. But long-term relationships within the scope of a specific healthcare reimbursement contract have restrictions. If the long-term co-operation does not prove worthwhile then the contracting partners must be able to terminate the relationship. This decision represents the ideal trade-off between the necessary flexibility and the internalisation of hold-up effects. Health insurers or service providers who are credited with an option of exploitation can in turn present a contractual obligation to provide services more credibly if they themselves undertake specific investments, settlement payments or transfer payments. Even then a “first-best” solution is not to be expected. The recipient receives the payment but still does not undertake a specific investment [7].

#### Assignment of contractual decision-making power

The contracting parties are able to limit the problem of hold-up through allocation of property rights and rights of control. Instead of actually planning for renegotiation, the parties can also agree in advance that one party shall be granted the residual right to undertake adjustment measures in the case of unforeseen circumstances. In addition, expected renegotiations can be structured by the agreement and ex-ante concretization of procedures [22]. It is expedient if the contracting party who is granted the rights is the party whose specific investment is the most difficult to secure against exploitation contractually in advance [7].

## Discussion and Conclusion

It has become clear that the coordination and motivation problems cannot be solved at no-costs. The presented problems in the design of selective individual contracts represent a necessary but not a sufficient condition for further government intervention. A state or political failures have to be assumed continuously [23].

In the future selective individual contracts will be completed in a competitive procurement process. Potential provider are identified and invited to tender. In the sense of solution options of information asymmetries prior to concluding detailed bids will be requested (screening), certificates on evaluations and external quality management measures will be sifted (signaling), several agreements for the purpose of self-selection by the health insurance will be put forward (self-selection) and offers will be evaluated regarding the trustworthiness (reputation). So that market forces can produce efficient allocation on the market for healthcare reimbursement contracts, the care provider need to state how they plan to guarantee the desired quality at an appropriate price-performance ratio (cost-effectiveness) for a specific population over a given period before signing a contract. Health insurance companies and care providers must be able to organize this process. If the findings of the principal-agent theory and the solution options are implemented in the practice, the existing information asymmetries can be reduced and the objectives of the parties harmonized. If long-term cooperation relations should facilitate efficient cooperation, trust must be established between the parties.
